# Cancer Risk Associated with Alcohol and Tobacco Use: Focus on Upper Aero-digestive Tract and Liver

**Published:** 2006

**Authors:** Claudio Pelucchi, Silvano Gallus, Werner Garavello, Cristina Bosetti, Carlo La Vecchia

**Affiliations:** Claudio Pelucchi, Sc.D., is a researcher; Silvano Gallus, Sc.D., is a senior researcher; and Cristina Bosetti, Sc.D., is head of the Unit of Cancer Epidemiology; all are at the Istituto di Ricerche Farmacologiche “Mario Negri” in Milan, Italy. Werner Garavello, M.D., is a researcher at the Istituto di Ricerche Farmacologiche “Mario Negri” in Milan, Italy, and a researcher at the Clinica Otorinolaringoiatrica, Dipartimento di Neuroscienze e Tecnologie Biomediche, Università Milano-Bicocca in Monza, Italy. Carlo La Vecchia, M.D., is head of the Laboratory of General Epidemiology at the Istituto di Ricerche Farmacologiche “Mario Negri” and an associate professor in epidemiology at the Istituto di Statistica Medica e Biometria at the Università degli Studi di Milano, both in Milan, Italy

**Keywords:** Alcohol and tobacco, alcohol consumption, ethanol, smoking, tobacco use, multiple drug use, cancer, risk factors, relative risk, population-attributable risk, oral cancer, pharyngeal cancer, laryngeal cancer, esophageal cancer, liver cancer, hepatocellular carcinoma

## Abstract

Alcohol and tobacco, alone or in combination, are associated with an increased risk of various cancers, including those of the upper aero-digestive tract and liver. Both alcohol and tobacco use can increase the risk of cancer of the oral cavity and throat (pharynx), and their combined use has a multiplicative effect on risk. Moreover, those regions of the mouth and pharynx that are more directly exposed to alcohol or tobacco are more likely to be affected by cancer than other regions. A similar effect was found with respect to cancer of the voice box (larynx). For squamous cell carcinoma of the esophagus, alcohol and tobacco also appear to increase risk synergistically. With liver cancer, in contrast, alcohol consumption and tobacco use appear to be independent risk factors.

Both alcohol and tobacco use are associated with numerous adverse health consequences, including an increased risk of certain types of cancer. For example, epidemiological studies found that alcohol consumption can increase the risk for cancers of the upper aero-digestive tract, stomach, large bowel (i.e., colon and rectum), liver, and breast, with higher levels of consumption leading to greater increases in risk (Bagnardi et al. 2001). Similarly, tobacco use is associated with an elevated risk of lung cancer, as well as of cancers of the upper aero-digestive tract, bladder, kidney, pancreas, stomach, and cervix and a certain type of leukemia (International Agency for Research on Cancer [IARC] 2004).

Many people use and abuse both alcohol and tobacco, and their combined effects on cancer risk also have been widely investigated. This article summarizes those findings, focusing on cancers at sites that are most directly exposed during alcohol and tobacco consumption—that is, the upper aero-digestive tract (i.e., the oral cavity, throat [pharynx], voice box [larynx], and esophagus) and the liver.

## Oral and Pharyngeal Cancer

In developed countries, oral and pharyngeal cancers rarely occur in people who neither smoke nor drink alcohol. However, many epidemiological studies conducted over the last three decades in the Americas, Europe, and Asia have provided strong evidence of an association between alcohol and tobacco use (both separately and in combination) and an increased risk of oral and pharyngeal tumors (Blot et al. 1988; Franceschi et al. 1990; Zheng et al. 1990, 2004).

### Risk Associated With Alcohol Consumption

The risk of both oral and pharyngeal cancer rises steeply with the level of alcohol consumption. An analysis that pooled data (i.e., a meta-analysis) from 26 studies of oral and pharyngeal cancers found that consumption of 25, 50, or 100 g pure alcohol/day^1^ was associated with a pooled relative risk (RR) of 1.75, 2.85, and 6.01, respectively, of oral and pharyngeal cancer (see Table 1) (Bagnardi et al. 2001). The RR indicates the strength of the relationship between a variable (e.g., alcohol consumption) and a given disease or type of cancer. People without the exposure (e.g., nondrinkers) are assigned a RR of 1.0. A RR greater than 1.0 indicates that the variable (e.g., drinking) increases the risk for that disease; furthermore, the greater the RR, the greater the association. Thus, the meta-analysis clearly demonstrated that the RR for oral or pharyngeal cancer increased significantly with increasing amounts of alcohol consumed. Similarly, another study conducted in Switzerland and Italy found that nonsmokers who consumed five or more drinks per day had a five-fold increased risk of these cancers compared with nondrinkers (Talamini et al. 1998).

The relationship between duration of alcohol consumption and risk of oral or pharyngeal cancer is less consistent. Moreover, the effect of drinking cessation on the RR for oral or pharyngeal cancer is unclear. Thus, it appears that the RR for these types of cancer appreciably declines only after 15 to 20 years of abstinence (Hayes et al. 1999).

Several studies also evaluated the effects of different types of alcoholic beverages on cancer risk. These analyses found that cancer risk generally was increased regardless of the type of beverage consumed. Moreover, the magnitude of the association between different types of beverages and cancer risk was inconsistent across studies and populations. In general, the beverage most frequently consumed in a population was associated with the highest risk of oral and pharyngeal cancer in that population (Boffetta and Hashibe 2006).

Studies conducted in animals have demonstrated that alcohol itself (i.e., ethanol) does not cause tumor development (i.e., is not carcinogenic). Instead, the primary breakdown product of ethanol in the body, acetaldehyde, has been shown to cause damage to the organism’s genetic material, the DNA, thereby contributing to cancer risk (Boffetta and Hashibe 2006). Additional studies found that those anatomic sites that come into closest contact with the ingested alcohol—that is, parts of the tongue and the region at the lower back of the throat (i.e., the hypopharynx)—are at highest risk of being affected by cancer (see Figure). In contrast, no association was found between alcohol consumption and an increased risk of cancer in the upper portion of the throat (i.e., the nasopharynx) and the salivary glands that are embedded into the wall of the oral cavity and throat (Boffetta and Hashibe 2006).

### Risk Associated With Smoking

The risk of oral and pharyngeal cancer also is strongly related to smoking. For example, the study conducted in Italy and Switzerland mentioned earlier also demonstrated that nondrinkers who smoked 25 or more cigarettes per day had a seven-fold increased risk of oral and pharyngeal cancer compared with nonsmokers (Talamini et al. 1998).

**Figure:**
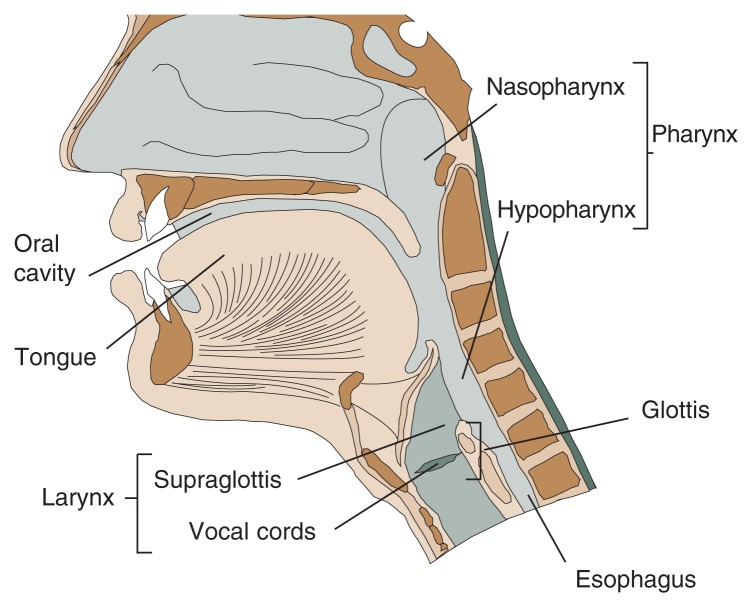
Anatomy of the upper aero-digestive tract.

Other studies found that the risk of these types of cancer increases with amount and duration of smoking, with duration of smoking having a greater impact on risk than amount. In addition, the risk of these cancers is higher in current smokers than in ex-smokers and is higher in people who start smoking at an earlier age than in people who start smoking at a later age (IARC 2004). An Italian study found that the RRs for oral cancer were 5.3 for people who smoked less than 15 cigarettes per day and 14.3 for people who smoked 25 or more cigarettes per day, compared with people who had never smoked (Franceschi et al. 1990). Furthermore, the RRs were 5.9 for people who had smoked for less than 30 years and 18.0 for people who had smoked for 40 years or more. Finally, the RR was higher (13.6) for people who had begun smoking before age 17.

The risk of oral and pharyngeal cancer is increased in smokers of all tobacco products, with a higher risk found in smokers of cigarettes without filters than in smokers of cigarettes with filters. In addition, some studies observed a stronger association between smoking and cancer in smokers of pipes and cigars than in smokers of cigarettes (Zheng et al. 2004). Similarly, in the Italian study mentioned above, the RR for oral cancer was 11.1 for cigarette smokers and 20.7 for pipe or cigar smokers (Franceschi et al. 1990).

### Risk Associated With Combined Alcohol Use and Smoking

The effect of combined exposure to alcohol and tobacco on risk of oral and pharyngeal cancer appears to be multiplicative—that is, the risk of combined exposure is the product of the increases in risk associated with exposure to either habit. Some studies found even greater (i.e., supra-multiplicative) increases in risk associated with combined exposure to alcohol and tobacco. For example, Zheng and colleagues (2004) demonstrated that people who drank heavily and smoked had a 300-times higher risk of these cancers than people who neither drank nor smoked.

The rate of oral cancer is particularly high among both men and women in South Asia. In this region, tobacco smoking often is replaced by or combined with chewing of tobacco and betel quid, which is another major risk factor for oral cancer (Parkin et al. 2005). Therefore, the role of tobacco smoking in combination with drinking in the development of oral cancer in Asia differs from that in Europe and North America.

Researchers also have assessed the role of smoking and drinking in causing oral and pharyngeal cancer in terms of population-attributable risks (PAR)—the portion of cases of a disease (e.g., oral cancer) in a population that is attributable to exposure to a risk factor (e.g., smoking or drinking). In other words, the PAR represents the proportion of cases of the disease that could be prevented if the risk factor were eliminated. PAR analyses determined that approximately 80 percent of oral and pharyngeal cancer cases in men and about 65 percent of cases in women can be attributed to alcohol and tobacco use (Blot et al. 1988; Negri et al. 1993; Hayes et al. 1999; Bosetti et al. 2000).

## Laryngeal Cancer

### Risk Associated With Alcohol Consumption

Both alcohol consumption and smoking are major risk factors for laryngeal cancer (IARC 2004; Altieri et al. 2005). Several studies have assessed the association of alcohol consumption with laryngeal cancer. These studies found that even in people who do not smoke, drinking is directly associated with the risk of laryngeal cancer, with risk increasing with the level of alcohol consumption (Altieri et al. 2005). A meta-analysis of 20 case-control studies^2^ of laryngeal cancer that together included more than 3,500 cases reported a strong direct relationship between alcohol consumption and risk of laryngeal cancer. Thus, people who consumed 25, 50, or 100 g of alcohol/day had RRs of 1.38, 1.94, and 3.95, respectively (see Table) (Bagnardi et al. 2001).

As with oral and pharyngeal cancer, additional studies assessing the role of different alcoholic beverages found that the most frequently consumed beverage in a population was associated with the highest risk of laryngeal cancer in that population. This finding indicates that it is the ethanol contained in all alcoholic beverages that determines the risk of laryngeal cancer rather than other compounds found in only some alcoholic beverages (Altieri et al. 2005).

### Risk Associated With Smoking

Case-control and cohort studies^3^ have consistently reported an elevated risk of laryngeal cancer in current smokers compared with people who have never smoked. Moreover, these studies found that the increase in RR was directly related to the number of cigarettes smoked and duration of smoking (IARC 2004). For example, the RR for laryngeal cancer was greater than 10 for smokers who had smoked for more than 40 years or for smokers of more than 20 cigarettes per day. Other studies determined that the RR rapidly declines after cessation of smoking and that this decline is greater the longer a person has stopped smoking. Thus, the risk of laryngeal cancer is reduced by about 60 percent in people who have stopped smoking for 10 to 15 years and is reduced even further in people who have stopped smoking for 20 years or more (Bosetti et al. 2006).

### Risk Associated With Combined Alcohol Use and Smoking

Several investigations have examined the combined effect of tobacco and alcohol use on the risk for laryngeal cancer (Altieri et al. 2005). The estimated RRs associated with the highest consumption levels of both alcohol and tobacco in those studies ranged from 8.0 to more than 100, suggesting that alcohol and tobacco consumption likely have multiplicative effects on risk.

Epidemiological studies that analyzed the influence of smoking and alcohol consumption on cancer development at various sites of the larynx found that the risk was higher for cancer in the area of the larynx which is located above the vocal cords and closest to the throat (i.e., the supraglottis) than in the area around the vocal cords (i.e., the glottis) (see Figure). This observation suggests that the risk of laryngeal cancer is highest in those areas of the larynx that come into closest contact with alcohol and tobacco smoke (IARC 2004; Altieri et al. 2005).

Researchers also determined the PAR for laryngeal cancer that is associated with alcohol and tobacco use. In an Italian study, about 25 percent of laryngeal cancer cases in men were found to be attributable to alcohol consumption and about 75 percent of cases were attributable to smoking (Tavani et al 1994).

### Esophageal Cancer

There are two main types of esophageal cancer: squamous cell carcinoma (SCC) and adenocarcinoma.^4^ The occurrence of esophageal adenocarcinomas is rapidly increasing in most developed countries. Studies found that tobacco and alcohol consumption are the dominant risk factors for SCC of the esophagus. The risk of esophageal adenocarcinoma, in contrast, is related to tobacco smoking (with a RR of 2 to 4) but not to alcohol drinking (Enzinger and Mayer 2003).

### Risk Associated With Alcohol Consumption

In populations with overall heavy alcohol consumption (e.g., northern France or Italy), the risk of esophageal SCC increases more strongly with the level of alcohol consumption than with the level of tobacco use—that is, people with the highest levels of alcohol consumption are at greater risk of esophageal SCC than people with the highest levels of smoking. Furthermore, the RR for esophageal SCC rapidly increases with the amount of alcohol consumed, whereas no consistent association exists between duration of alcohol use or age at initiation of alcohol use and SCC risk (Franceschi et al. 1990).

As with oral, pharyngeal, and laryngeal cancer, the most frequently consumed alcoholic beverage in a population tends to be associated with the highest risk of esophageal cancer, although some studies have suggested that the risk is higher in people who consume beverages with a higher alcohol content (Castellsagué et al. 1999).

### Risk Associated With Smoking

In contrast to alcohol consumption, both duration and daily amount of smoking are major determinants of esophageal cancer risk associated with tobacco use (Franceschi et al. 1990). Comparisons of cancer risk associated with different tobacco products found that use of products containing black tobacco (which contains higher levels of chemicals known as N-nitroso compounds) may be associated with a higher risk of esophageal SCC than use of products containing blond tobacco (Castellsagué et al. 1999). Furthermore, smoking of pipes and cigars increases the risk of esophageal cancer at least as much as does cigarette smoking.

### Risk Associated With Combined Alcohol Use and Smoking

Alcohol and tobacco act with a multiplicative effect on the risk of esophageal SCC. In a study involving three regions of the United States, the RR for combined heavy alcohol and tobacco use was 35.4 in white males and 149.2 in black males, compared with men of those racial groups who were non- or light smokers and drinkers (Brown et al. 1994).

In terms of PAR, alcohol and tobacco consumption are responsible for more than 80 percent of esophageal SCC cases in Europe and the Americas. Additional analyses found that the PAR was higher in men (about 90 percent) than in women (between 30 and 50 percent) (Castellsagué et al. 1999). Moreover, the PAR was higher in black men (93 percent) than in white men (86 percent) in the United States (Brown et al. 1994). These gender and racial differences in alcohol- and tobacco-related PAR account for nearly all of the observed differences in the incidence of esophageal SCC between men and women and between black and white American men.

## Liver Cancer

Liver cancer, or hepatocellular carcinoma (HCC), is globally the sixth most common cancer and the third most common cause of cancer death (Parkin et al. 2005). In the developed countries, HCC is relatively rare compared with the developing world, although during the past decade the incidence of primary liver cancer^5^ has strongly increased in the United States (Howe et al. 2001) and in several European countries (La Vecchia et al. 2000). One risk factor for the development of HCC is chronic infection with hepatitis B virus (HBV) or hepatitis C virus (HCV), which increases the risk of HCC by approximately 20-fold (Parkin et al. 2005).

### Risk Associated With Alcohol Consumption

Most cases of HCC occur in patients with cirrhosis of the liver, which often occurs as a consequence of long-term heavy alcohol consumption. In fact, development of liver cirrhosis appears to be an important step in the development (i.e., pathogenesis) of liver cancer (London and McGlynn 1996).

Several studies have demonstrated that alcohol consumption increases the risk of HCC. A meta-analysis of 20 studies of liver cancer that included 2,294 cases of HCC reported a direct trend in risk with increasing alcohol consumption (Bagnardi et al. 2001). Thus, the RR was 1.17 with consumption of 25 g of alcohol/day, 1.36 with consumption of 50 g/day, and 1.86 with consumption of 100 g/day (see Table). It is likely, however, that the associations found in epidemiological studies still underestimate the risk associated with alcohol consumption, because in most cases alcoholrelated HCC develops in people who are already suffering from cirrhosis and have already reduced their alcohol consumption because of the cirrhosis (Bagnardi et al. 2001).

### Risk Associated With Smoking

Many studies have examined the association between tobacco smoking and the development of HCC. A review of data from 30 cohort and 30 case-control studies concluded that almost all cohort studies and several case-control studies demonstrated a direct association between smoking and HCC risk (IARC 2004). In most studies the overall RR for current smokers (compared with people who had never smoked) was between 1.2 and 2.0. Moreover, the RR was found to be higher for heavy smokers than for light smokers.

Additional studies indicated that the increase in HCC risk associated with cigarette smoking appears to be greater among people infected with HBV and HCV than in people not infected with these viruses. However, this relationship was demonstrated only for geographical regions with a high incidence of HCC (IARC 2004; Wang et al. 2003; Fujita et al. 2006) but not in regions with a low incidence (Kuper et al. 2000).

### Risk Associated With Combined Alcohol Use and Smoking

Tobacco and alcohol consumption are positively correlated—that is, people who drink are more likely to smoke than nondrinkers, and vice versa. As a result, it is difficult to separate the effects of alcohol consumption and smoking on HCC risk. For example, estimates of the role of tobacco as a risk factor may be influenced by the above-mentioned fact that people who develop cirrhosis prior to HCC may already have reduced their alcohol consumption. This would result in models that are underadjusted for alcohol use and which consequently result in an overestimation of the RRs of HCC for smoking. These difficulties notwithstanding, an interaction between alcohol drinking and tobacco smoking was found in two case-control studies from Greece (Kuper et al. 2000) and the United States (Marrero et al. 2005).

The studies conducted to date indicate that alcohol consumption and smoking are independent risk factors for HCC. How large the RR associated with these two habits is, however, remains the subject of discussion.

Researchers also have conducted PAR analyses of the influence of drinking and smoking on HCC risk. A Korean study estimated that about 25 percent of HCC cases are attributable to tobacco smoking and about 5 percent of cases are attributable to alcohol consumption (Jee et al. 2004). Because of the above-mentioned reduction in drinking levels in people with cirrhosis, however, the PAR associated with alcohol consumption likely is underestimated in this study. Another PAR analysis conducted in Taiwan found that alcohol consumption, betel quid chewing, and cigarette smoking accounted for 25 percent of all HCC cases. Among people infected with HBV, approximately 30 percent of HCC cases could be attributed to the use of alcohol and/or tobacco (Wang et al. 2003).

**At a Glance**

**Effects of Alcohol Consumption and Smoking on Cancer Risk**

**Oral cancer**

- People who drank heavily and smoked had a 300-times higher risk of oral and pharyngeal cancer than people who neither drank nor smoked (Zheng et al. 2004).
- Approximately 80 percent of oral and pharyngeal cancer cases in men and about 65 percent of cases in women can be attributed to alcohol and tobacco use (Blot et al. 1988; Negri et al. 1993; Hayes et al. 1999; Bosetti et al. 2000).

**Laryngeal cancer**

- In various studies, the relative risk (RR) of laryngeal cancer associated with the highest consumption levels of both alcohol and tobacco ranged from 8.0 to more than 100 (Altieri et al. 2005).
- In an Italian study, 25 percent of laryngeal cancer cases in men could be attributed to alcohol consumption and about 75 percent could be attributed to smoking (Tavani et al. 1994).

**Esophageal cancer**

- In a U.S. study, the RR for esophageal squamous cell carcinoma (SCC) associated with combined heavy alcohol and tobacco use was 35.4 in White males and 149.2 in Black males compared with non- or light smokers and drinkers (Brown 1994).
- More than 80 percent of esophageal SCC can be attributed to alcohol and tobacco consumption (Castellsagué et al. 1999; Brown et al. 1994).

**Liver cancer**

- Alcohol consumption and smoking appear to be independent risk factors for hepatocellular carcinoma (HCC); the exact relative risks associated with both habits are unknown.
- In several studies, between 25 and 30 percent of HCC cases could be attributed to alcohol and/or tobacco use (Jee et al. 2004; Wang et al. 2003).

## Summary

Alcohol consumption and smoking are major risk factors for cancers of the upper aero-digestive tract, accounting for approximately three-quarters of cases in developed countries. For alcohol consumption, the level of consumption determines risk to a greater degree than does duration of consumption. For smoking, in contrast, level and duration of smoking have similar impacts on the risk of upper aero-digestive tract cancers. Combined exposure to alcohol and tobacco has a multiplicative effect on the development of these types of cancer.

Both alcohol and tobacco consumption also are causally related to liver cancer; however, these associations are only moderate and a smaller fraction of liver tumors are attributable to these factors compared with cancers of the upper aero-digestive tract. Some studies have reported that an interaction between alcohol consumption and smoking exists with respect to HCC risk, but this issue has not yet been fully explored.

## Figures and Tables

**Table:** Association Between Level of Alcohol Consumption and the Development of Certain Types of Cancer

Type of Cancer	Pooled RR (95% Confidence Interval) Associated With Alcohol Consumption*		

	25 g/day	50 g/day	100 g/day
Oral and Pharyngeal Cancer	1.75 (1.70–1.82)	2.85 (2.70–3.04)	6.01 (5.46–6.62)
Laryngeal Cancer	1.38 (1.32–1.45)	1.94 (1.78–2.11)	3.95 (3.43–4.75)
Esophageal Cancer	1.51 (1.48–1.55)	2.21 (2.11–2.31)	4.23 (3.91–4.59)
Liver Cancer	1.17 (1.11–1.23)	1.36 (1.23–1.51)	1.86 (1.53–2.27)

* The consumption levels analyzed correspond to approximately two, four, and eight standard drinks per day, respectively. A standard drink is frequently defined as 12 fl oz of beer, 5 fl oz of wine, or 1.5 fl oz of 80-proof distilled spirits, all of which contain approximately 0.5 oz (14 g) of pure alcohol.

SOURCE: Bagnardi et al. 2001

## Footnotes

1 In the United States, a standard drink is frequently defined as 0.5 ounces (oz) or 14 grams of pure alcohol. This alcohol amount is contained in 12 fluid oz of beer, 5 fluid oz of wine, or 1.5 fluid oz of 80-proof distilled spirits. Thus, the consumption levels analyzed here correspond to approximately two, four, and eight standard drinks per day, respectively.

2 Case-control studies compare people with a certain disease (e.g., laryngeal cancer)—the cases—with a similar group of people without the disease—the controls. For both cases and controls, investigators collect information on potential risk factors (e.g., smoking and alcohol consumption) to determine whether significant differences exist between the two groups.

3 Cohort studies follow groups of initially healthy people over an extended period, monitoring disease development over time. At the outset and throughout the study, investigators determine participants’ risk factors (e.g., level of drinking or smoking) so that they can relate these risk factors with disease development during followup.

4 Squamous cell carcinomas develop, as the name implies, in a cell type called squamous cells, which line the inner surface of the esophagus. Adenocarcinomas originate in glandular tissue. Although the esophagus normally does not contain glandular cells, some squamous cells, particularly in the lower esophagus, may be replaced with glandular cell, which may develop into a tumor.

5 The term “primary liver cancer” refers to tumors that initially develop in the liver before possibly spreading to other organs or tissues. In many people, however, cancer first develops in other organs or tissues and only later spreads to the liver to cause liver cancer.
